# Supplementary material for: Risks and Benefits of Judo Training for Middle-Aged and Older People: A Systematic Review
Source: Sports (Basel). 2023 Mar 14;11(3):68. doi: 10.3390/sports11030068 (PMC10058523; doi:10.3390/sports11030068)
Supplement: Supplementary file 1 [file sports-11-00068-s001.zip › Supplementary Material 1. modified Rev_1.pdf]

Supplementary Materials ~~1~~. Study quality and risk of bias (Code refers to full reference available in Table 1).

Code	A	B	C	D	E	F	G	Low	Moderate	Serious	Critical	NI	Rating*
35	Serious	Low	Low	Low	NI	Moderate	Moderate	3	2	1	0	1	**Serious**
36	Serious	Low	Low	Low	NI	Moderate	Moderate	3	2	1	0	1	**Serious**
37	Serious	Low	Low	Low	NI	Moderate	Moderate	3	2	1	0	1	**Serious**
38	Moderate	Low	Low	Low	Low	Moderate	Moderate	3	2	1	0	0	**Moderate**
39	Serious	Low	Low	Moderate	NI	Moderate	Moderate	2	3	1	0	1	**Serious**
40	Serious	Low	Low	Low	NI	Moderate	Moderate	3	2	1	0	1	**Serious**
41	Moderate	Low	Low	Low	Low	Moderate	Moderate	4	3	0	0	0	**Moderate**
42	Moderate	Low	Low	Low	Low	Moderate	Moderate	4	3	0	0	0	**Moderate**
43	Moderate	Low	Low	Low	NI	Moderate	Moderate	3	3	0	0	1	**Serious**
44	Serious	Low	Low	Low	Low	Low	Moderate	4	1	1	0	0	**Serious**

Note: ROBINS-I tool (Sterne et al., 2016) checklist items for *non-randomised controlled studies* related to bias in the following domains: **A**. Confounders; **B**. Participants selection; **C**. Classification of interventions; **D**. Deviations from intervention; **E**. Missing data; **F**. Outcomes measurement; **G**. Results selection. NI=No information.

* Rating: Declaring a study to be at a particular level of risk of bias for an individual domain will mean that the study as a whole has a risk of bias at least this severe.

Code	A	B	C	D	E	F	G	H	I	J	K	L	M	N	YES	NO	CD	NA	Rating#
45	Yes	Yes	No	Yes	No	No	Yes	Yes	Yes	No	No	No	NA	No	**6**	**7**	0	1	Fair
46	Yes	Yes	Yes	No	No	No	Yes	Yes	Yes	No	Yes	No	NA	No	**7**	**6**	0	1	Fair
47	Yes	Yes	No	NA	No	No	Yes	Yes	Yes	No	Yes	No	NA	No	**6**	**6**	0	2	Fair
48	Yes	Yes	Yes	No	No	No	Yes	Yes	Yes	No	Yes	No	NA	No	**7**	**6**	0	1	Fair
49	Yes	Yes	Yes	No	No	No	Yes	Yes	Yes	No	Yes	No	NA	No	**7**	**6**	0	1	Fair
50	Yes	Yes	Yes	No	No	No	Yes	Yes	Yes	No	Yes	No	NA	Yes	**8**	**5**	0	1	Fair
51	Yes	Yes	Yes	CD	No	No	CD	Yes	Yes	No	Yes	No	NA	No	**6**	**5**	2	1	Fair

Note: NIH tool (https://www.nhlbi.nih.gov/health-topics) checklist items for *cross-sectional studies* related to quality in: **A**. Research question; **B** and **C**. Study population; **D**. Eligibility criteria; **E**. Sample size justification; **F**. Exposure assessment time; **G**. Sufficient timeframe to see an effect; **H**. Levels of exposure; **I**. Exposure measures; **J**. Repeated exposure assessment; **K**. Outcome measures; **L**. Blinding of outcome assessors; **M**. Follow-up rate; and **N**. Statistical analyses. CD=Cannot determine; NA=Not Applicable.

Rating: Good = 10–14 yes, Fair = 5–9 yes, or Poor = 0–4 yes.

Code	A	B	C-D	E	F	G	H	I	J-K-L	M	N	O	P	Q	R	S	T	U	V	W	YES	NO	CD	Rating§
52	Yes	Yes	Yes	No	Yes	Yes	No	No	Yes	Yes	No	Yes	Yes	Yes	Yes	No	No	No	Yes	Yes	**16**	7	0	**Fair**
53	Yes	Yes	Yes	No	No	No	No	Yes	Yes	Yes	No	Yes	Yes	Yes	Yes	No	No	Yes	CD	CD	**14**	7	2	**Fair**
54	Yes	Yes	Yes	No	Yes	Yes	No	No	Yes	CD	No	Yes	Yes	Yes	No	No	No	No	CD	CD	**12**	8	3	**Fair**
55	Yes	Yes	Yes	Yes	No	Yes	Yes	No	Yes	Yes	No	Yes	Yes	Yes	Yes	No	Yes	No	Yes	Yes	**18**	5	0	**Good**
56	Yes	Yes	Yes	Yes	No	Yes	Yes	Yes	Yes	Yes	No	Yes	No	Yes	Yes	No	No	Yes	Yes	Yes	**18**	5	0	**Good**
57	Yes	No	Yes	Yes	No	Yes	No	Yes	Yes	CD	No	Yes	No	No	No	No	No	No	CD	CD	**10**	10	3	**Fair**

Note: AGREE II tool (Brouwers et al., 2010) checklist items for *methodological studies* related to quality in six domains: **A**, **B** and **C**. Scope and purpose; **D**, **E**, **F**, **G**. Stakeholder involvement; **H**, **I**, **J**, **K**, **L**, **M**, **N**. Rigor of development; **O**, **P**, **Q**, **R**. Clarity of presentation; **S**, **T**, **U**. Applicability; **V**, **W**. Editorial independence. CD=Cannot Determine. In the table, items C and D, and J, K and L have been aggregated because all studies received the same evaluation.

§ Rating: Good = 16–23 yes, Fair = 8–15 yes, or Poor = 0–7 yes.
